# Analysis of choroidal thickness in juvenile systemic lupus erythematosus and its correlation with laboratory tests

**DOI:** 10.1186/s12886-023-02906-4

**Published:** 2023-04-11

**Authors:** Li Ru, Jinping Xu, Zhongjing Lin, Lanfang Cao, Lin Zhang

**Affiliations:** 1 Department of Ophthalmology, Renji Hospital, School of Medicine, Shanghai Jiaotong University, Shanghai, China; 2 Department of Pediatrics, Renji Hospital, School of Medicine, Shanghai Jiaotong University, Shanghai, China

**Keywords:** Choroidal thickness, Systemic lupus erythematosus, Optical coherence tomography, Cytokine

## Abstract

**Background:**

The aim of this study is to investigate the alterations of choroidal thickness (CT) in juvenile systemic lupus erythematosus (JSLE) using enhanced depth imaging optical coherence tomography (EDI-OCT). We also aimed to assess whether CT parameters correlated with systemic health status in JSLE patients.

**Methods:**

JSLE patients and age- and sex-matched healthy subjects were recruited. A detailed ophthalmological examination was applied to all participants. CT measurements were acquired in the macular region using EDI-OCT. Moreover, a spectrum of laboratory tests was examined to evaluate the systemic conditions, and the Th1/Th2/Th17/Treg cytokine profiles in the peripheral blood were also analyzed in JSLE group.

**Results:**

A total of 45 JSLE patients with no visual impairment and 50 healthy individuals were enrolled in the study. CT values in the macular region were decreased in JSLE patients when compared with healthy controls, even adjusting for age, axial length and refraction. There were no significant correlations between CT and cumulative dose of hydroxychloroquine or duration of hydroxychloroquine use (all P > 0.05). The average macular, temporal and subfoveal CT in JSLE group was negatively correlated with IL-6 and IL-10 (all P < 0.05), but had no significant correlations with other laboratory results (all P > 0.05).

**Conclusions:**

JSLE patients without ocular involvement may have significant variations in choroidal thickness at the macular area. Choroidal alterations might be associated with the systemic cytokine profiles in JSLE.

## Background

Systemic lupus erythematosus (SLE) is a prototypical complex autoimmune disease, which is featured by a large array of autoantibody productions. It occurs more commonly in females than males. The clinical presentation varies widely among patients and can change throughout the disease course, making its early diagnosis challenging. The disease can affect multiple organs with varying degrees of severity, involving renal, dermatologic, pulmonary, cardiac, musculoskeletal, and ocular manifestations [1, 2].

Ocular involvement is not a rare entity at the initial diagnosis of SLE, approximately affecting 10% of SLE patients. Ocular variations also correlate with systemic disease activity. SLE patients can present with a myriad of ocular manifestations, such as orbital disorders, keratoconjunctivitis, scleritis, episcleritis, retinopathy, choroidopathy, optic neuritis [3–6]. The most visually-threatening complications are related to lupus retinopathy and choroidopathy. Ischemic optic neuropathy, retinal vein and artery occlusion caused by thrombogenesis have been reported in SLE patients [7]. The choroid is mainly composed of blood vessels and pigment cells, and it serves nutrition to the retinal photoreceptors. It has been implicated in the pathogenesis of various ocular inflammatory disorders [8, 9]. With fundus fluorescein angiography (FFA) and indocyanine green angiography (ICGA), a spectrum of vascular abnormalities was identified, even if SLE patients showed normal funduscopic appearance, including focal fluorescein leakage, peripheral capillary dropout and nonperfusion, delayed filling of choroidal lobules, thinning of choroidal capillaries [10–12]. However, invasive methods will limit their clinical applications to some extent, especially in children.

Optical coherence tomography (OCT) is a promising imaging technique that is capable of imaging the morphologic changes of the choroid noninvasively. With the advancement in technology, enhanced depth imaging (EDI) mode has promoted an increase in quantifying choroidal thickness (CT) in various populations [13]. However, recent studies in the literature put more emphasis on the evaluation of CT in adult SLE patients. Less is known about how the choroid could be affected in the pediatric SLE population. Juvenile SLE (JSLE) is associated with more severe presentations and higher morbidity and mortality, requiring more aggressive treatment strategies compared to adult-onset SLE [14–16]. Considering up to 20% SLE patients were initially diagnosed in childhood and adolescence [17], it is necessary to investigate the alterations of CT in JSLE individuals. Therefore, in this preliminary research, we aimed to evaluate the choroid changes in JSLE patients without clinical ocular manifestations. As JSLE patients often present with systemic damage, and persistently elevated levels of plasma cytokines have been identified, data on the links between CT parameters and systemic alterations measured by various laboratory tests has not been well established. Thus, we also aimed to assess whether CT correlated with systemic health status, which might provide adjunctive information for the clinical evaluation and monitoring.

## Materials and methods

### Study population

The study was approved by the Ethics Committee of Renji Hospital, School of Medicine, Shanghai Jiaotong University, and it was conducted according to the Declaration of Helsinki. The study was performed with the cooperation between the departments of rheumatology and ophthalmology from January 2019 to December 2020. Permissions for publications of clinical data were acquired from all children’s guardians at the time of enrollment.

Consecutive JSLE participants were recruited from the department of rheumatology who sought for ophthalmological screening. The children were diagnosed with JSLE according to 2012 Systemic Lupus International Collaborating (SLICC) classification criteria [18]. According to the scoring system of Systemic Lupus Erythematosus Disease Activity Index 2000 (SLEDAI-2 K) [19], patients with a score of 0–4 are basically considered to be in remission without activity in the major organ system, a score of 5–9 is regarded as mild activity, a score of 10–14 means moderate activity, and ≥ 15 refers to severe activity. All enrolled children were with a score ≤ 4 over the past 10 days. In other words, they were classified into the inactive phase. JSLE patients in the active phase or with ocular involvement were not involved in the current study. The duration of SLE diagnosis was also recorded. The control group were age- and sex-matched healthy individuals who sought for vision screening and volunteered to participate in the study. The overall exclusion criteria were listed as follows: a refractive error > 6 diopters, astigmatism > 3 diopters, additional systemic diseases (such as hypertension, diabetes mellitus, chronic renal failure), ocular disease (such as cataract, glaucoma, uveitis, retinopathy, choroidopathy), history of ocular trauma and surgery.

### Ophthalmological examination

Each participant underwent a spectrum of ophthalmic examinations, involving best-corrected visual acuity (BCVA) (Snellen visual acuity was transformed to logMAR equivalents), refraction measured using NIDEK ARK-510 A, intraocular pressure (IOP) measurement using auto noncontact tonometer (TX-F, Canon, Tokyo, Japan), slit lamp examination and fundus examination. Central corneal thickness (CCT) was measured by ultrasound pachymetry. Axial length was recorded using IOL-Master (Carl Zeiss Meditec, Jena, Germany).

### OCT measurements

All participants were imaged using spectral domain OCT (SD-OCT) (Spectralis; Heidelberg Engineering, Heidelberg, Germany). One experienced operator, who was blinded in terms of groups, was invited to capture the images. Only images with signal strength index > 8 were recorded for further analysis. To improve the visualization of the choroid, OCT scan with EDI mode was performed using 6-mm line across the fovea. All scans were performed in the same time frame from 1 pm to 4 pm to avoid potential diurnal variations. The same operator conducted all the measurements within one week to minimize the measurement bias. CT was measured from the outer boundary of the retinal pigment epithelium to the inner edge of choroid-sclera junction. If it is difficult to determine the boundary of the choroid-sclera junction, the study subjects would be excluded. We selected several determined measurement points at subfovea, 500 μm, 750 μm, 1000 μm, 1250 and 1500 μm both temporal and nasal to the fovea. The right eye was chosen for the final statistical analysis.

### Laboratory analysis

Various laboratory tests were examined to determine the systemic involvement and assess the disease activity in JSLE patients. Blood samples, after a 10-h overnight fast, were collected within one week after ophthalmologic examinations. The following data were acquired using standard laboratory methods: C-reactive protein (CRP), erythrocyte sedimentation rate (ESR), serum alanine transferase (ALT), aspartate aminotransferase (AST), serum creatinine (Scr), creatine kinase (CK), CK-myocardial band (CK-MB), complement 3 (C3), complement 4 (C4), anti-dsDNA antibody. A spectrum of cytokines expressing Th1/Th2/Th17/Treg pattern were also analyzed, including IL-2, IL-4, IL-6, IL-10, IFN-γ, TNF-α and IL-17 A.

### Statistical analysis

The Statistical Package for Social Sciences software version 26.0 (SPSS Inc, Chicago, IL, USA) was used for statistical analysis. The Shapiro-Wilk test was chosen to check the data distributions, while the Levene test was considered to test the variance homogeneity. Accordingly, quantitative data were summarized as mean ± standard deviation (SD) or median (interquartile range). The Chi-square test was used for comparisons of categorical variables. Independent samples t test was performed for variables with the normality assumption, otherwise, Mann-Whitney U test was selected. CT values were further compared using covariate analysis after adjusting for age, axial length and refraction. After checking the normality of data, correlations between different parameters were explored using the Pearson or Spearman correlation coefficient. P < 0.05 was regarded as statistically significant.

## Results

### Clinical characteristics of the study population

The main demographic and clinical characteristics of the study population are presented in Table 1. A total of 45 JSLE patients and 50 healthy subjects were enrolled in the current study. As for JSLE group, 32 were female (71.1%) and 13 were male (28.9%), with a mean age of 13.7 ± 2.8 years. The mean age at onset of disease was 10.7 ± 2.0 years. Of note, these JSLE patients were clinically stable, and the median SLEDAI score was 2 (range: 0–4). All JSLE patients at the enrollment were receiving hydroxychloroquine (HCQ) and OCT scans revealed no evidence of structural alterations favoring toxic retinopathy. The median cumulative dose of HCQ was 188.5 g (range: 38.4–576) and the median HCQ duration was 27 months (range: 6–68). In terms of sex distribution and age, no significant differences were observed as compared to the control group (P = 0.256, P = 0.096, respectively). There were also no significant differences in IOP, CCT, axial length and refraction between the two study groups (all P > 0.05). All study cases achieved BCVA of 20/20. None of JSLE cases showed any evidence of ocular involvement at the examination visit.

**Table 1:** Demographic and clinical characteristics of JSLE patients and normal controls

	JSLE group	Control group		P value
No. of subjects	45	50		
Sex (male/female)	13/32	20/30		0.256
Age (years)	13.7 ± 2.8	12.7 ± 2.8		0.096
IOP (mmHg)	15.2 ± 4.1	15.5 ± 2.9		0.696
CCT (µm)	524 ± 33	535 ± 30		0.112
Axial length(mm)	23.97 ± 1.08	24.00 ± 1.30		0.877
Refraction (D)	-1.63 ± 1.60	-1.29 ± 2.22		0.394

JSLE: juvenile systemic lupus erythematosus; IOP: intraocular pressure; CCT: central corneal thickness

### Laboratory parameters in JSLE patients

Table 2 summarizes the systemic laboratory findings in JSLE patients. The median level of CRP was 0.7 mg/L, and all subjects were within the normal range. The median level of ESR was 13.0 mm/h, with 37.8% patients exceeding the normal limits. The plasma levels of ALT, Scr, CK and CK-MB remained in the normal range, and only two patients had elevated AST. These results suggested that no obvious damage to hepatic, renal and myocardial functions exited in JSLE patients. The median plasma level of anti-dsDNA antibody was 31.2 IU/ml, and 30 (66.7%) patients showed elevated levels of anti-dsDNA antibody. Decreased levels of C3 and C4 existed in 44.4% and 35.6% JSLE patients, respectively. Th1/Th2/Th17/Treg related cytokines also changed in varying degrees in these JSLE patients, with IL-6 achieving the highest portion (40.0%).

**Table 2:** Systemic laboratory results in JSLE patients

	Reference values	Clinical values	Abnormality N (%)
CRP	0–10 mg/L	0.7 (0.4–2.35)	0 (0)
ESR	0–20 mm/h	13.0 (5.5–27.5)	17 (37.8)
ALT	0–75 U/L	20.0 (14.0-32.5)	0 (0)
AST	0–40 U/L	18.0 (15.5–24.5)	2 (4.4)
Scr	45–104 µmol/L	40.0 (35.0-43.8)	0 (0)
CK	38–174 U/L	36.0 (21.5–50.0)	0 (0)
CK-MB	0.0–25.0 U/L	13.0 (10.0–16.0)	0 (0)
ds-DNA	< 77 IU/ml	31.2 (24.3-184.9)	30 (66.7)
C3	0.9–1.8 g/L	0.94 (0.69–1.10)	20 (44.4)
C4	0.1–0.4 g/L	0.16 (0.07–0.21)	16 (35.6)
IL-2	0-5.71 pg/ml	1.13 (0.72–1.56)	0 (0)
IL-4	0-2.80 pg/ml	1.84 (1.04–2.78)	11 (24.4)
IL-6	0-5.30 pg/ml	4.22 (1.85–7.97)	18 (40.0)
IL-10	0-4.91 pg/ml	3.03 (1.94–5.14)	12 (26.7)
TNF-α	0-2.31 pg/ml	1.19 (0.86–1.89)	8 (17.8)
INF-γ	0-7.42 pg/ml	1.64 (1.05–2.69)	3 (6.7)
IL-17 A	0-20.6 pg/ml	4.18 (2.29–15.16)	7 (15.6)

Data were expressed as median (interquartile range)

CRP: C-reactive protein; ESR: erythrocyte sedimentation rate; ALT: alanine transferase; AST: aspartate aminotransferase; Scr: serum creatinine; CK: creatine kinase; CK-MB: creatine kinase-myocardial band; C3: complement 3; C4: complement 4

### OCT findings between JSLE patients and healthy controls

The parameters of CT in the macular region using the original data are shown in Table 3. The average macular CT in JSLE patients was significantly thinner than that in the healthy controls (220±41 μm vs. 259±56 μm, P < 0.001). The mean subfoveal CT was 235 ± 46 μm in JSLE patients and was 284 ± 60 μm in the control group (P < 0.001). Apart from this, CT was significantly thinner at all measurement points in JSLE patients compared to the control group (all p < 0.01). The comparison results remained the same even after adjusting for age, axial length and refraction (all p < 0.01). Figure 1 displayed the changes of CT at different locations with a horizon scan.

**Table 3:** Comparison of choroidal thickness at different measurement points in the macular area between JSLE group and control group

location	JSLE group	Control group	P *	P #
Average CT	220 ± 41	259 ± 56	< 0.001	< 0.001
Subfoveal CT	235 ± 46	284 ± 60	< 0.001	< 0.001
Average nasal CT	208 ± 42	243 ± 57	0.001	0.001
N 500	224 ± 45	266 ± 60	< 0.001	< 0.001
N 750	215 ± 43	256 ± 59	< 0.001	< 0.001
N 1000	208 ± 43	242 ± 57	0.002	0.003
N 1250	200 ± 41	231 ± 57	0.003	0.004
N 1500	193 ± 45	221 ± 56	0.009	0.013
Average temporal CT	229 ± 44	270 ± 58	< 0.001	0.001
T 500	232 ± 45	280 ± 61	< 0.001	< 0.001
T 750	232 ± 47	274 ± 59	< 0.001	< 0.001
T 1000	231 ± 45	270 ± 60	0.001	0.001
T 1250	227 ± 45	264 ± 60	0.001	0.002
T 1500	225 ± 46	261 ± 58	0.001	0.003

Quantitative data were expressed as mean ± SD.

CT: choroidal thickness; N, nasal to the fovea; T, temporal to the fovea

* Independent t-test comparing choroidal thickness between JSLE group and control group using the original data

# comparison of choroidal thickness between JSLE group and control group after adjusting for age, axial length and refraction using covariance analysis

**Fig. 1:**
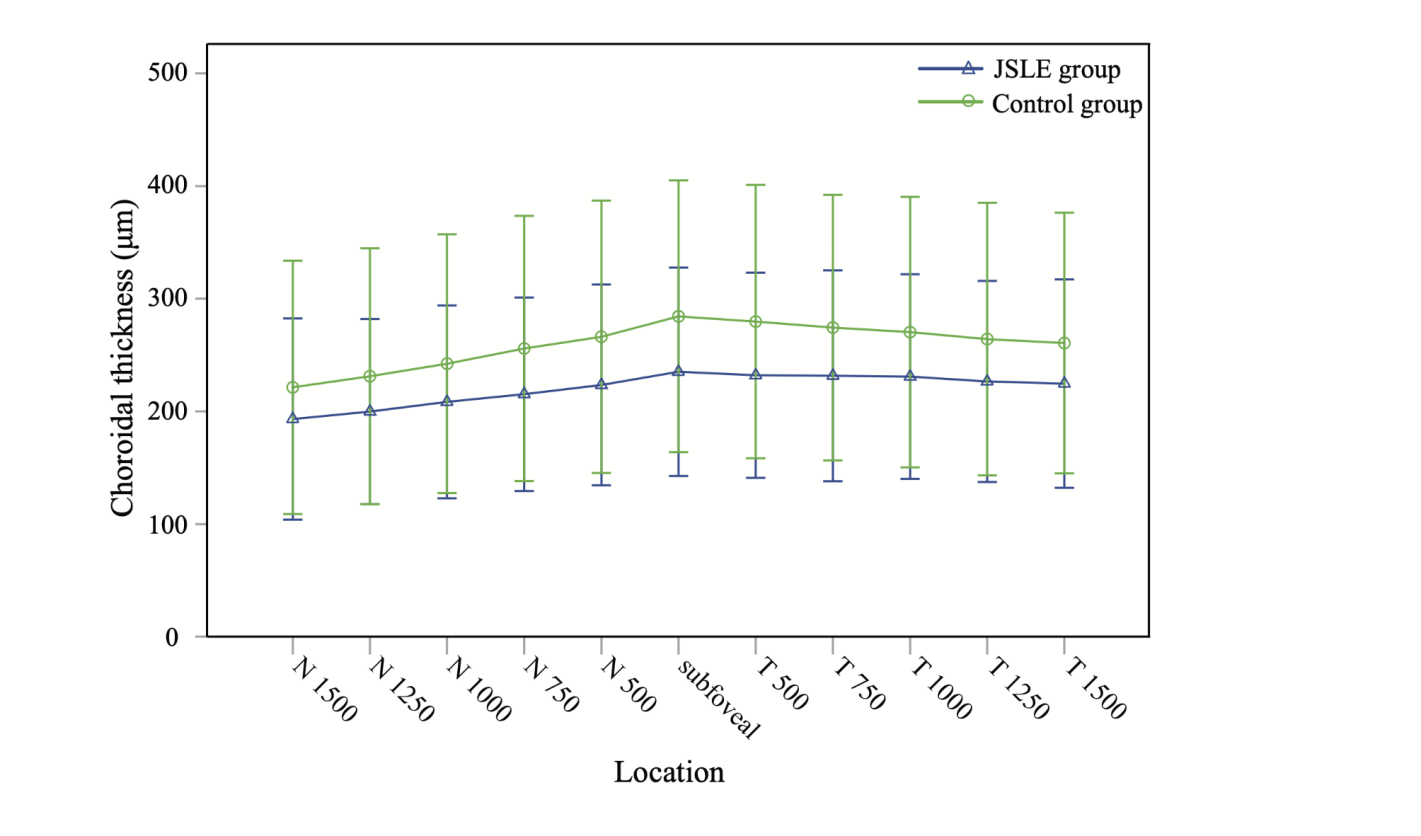
Graph showing comparison of choroidal thickness at different locations in the macular region with a horizon scan. The vertical bars represented standard deviation N, nasal to the fovea; T, temporal to the fovea

### The correlations between CT and clinical parameters

**Fig. 2:**
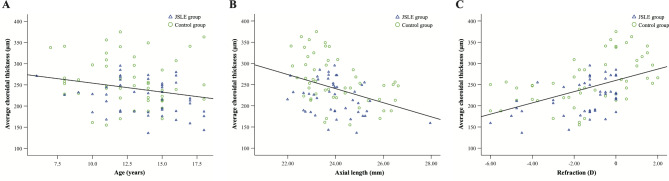
Scatter plots of choroidal thickness against age **(A)**, axial length **(B)** and refraction **(C)** in all participants

Correlation analysis was performed between CT parameters and clinical parameters in all participants (Table 4). Average macular CT presented negative associations with age (r=-0.225, P = 0.029) and axial length (r=-0.374, P < 0.001), while it was positively correlated with refraction (r = 0.487, P < 0.001). Figure 2 showed the scatter plots and correlation analysis of average macular CT against age, axial length, and refraction. Similar results were identified in the analysis of subfoveal, average temporal and nasal CT. All CT parameters showed no significant correlations with IOP and CCT (all P > 0.05). Spearman correlation analysis revealed no significant correlations between SLEDAI score and different CT parameters (all P > 0.05). In addition, these CT parameters had no significant relationships with cumulative HCQ dose or duration of HCQ use (all P > 0.05) (Table 5).

**Table 4:** The associations between choroidal thickness and clinical variables in all the participants

	average CT			subfoveal CT			average temporal CT			average nasal CT	
	r	P		r	P		r	P		r	P
age	-0.225	0.029*		-0.251	0.014*		-0.213	0.038*		-0.212	0.040*
IOP	-0.052	0.619		-0.038	0.712		-0.085	0.413		-0.017	0.870
CCT	-0.131	0.206		-0.094	0.365		-0.138	0.182		-0.124	0.233
axial length	-0.374	< 0.001#		-0.394	< 0.001#		-0.320	0.002&		-0.396	< 0.001#
refraction	0.487	< 0.001#		0.490	< 0.001#		0.429	< 0.001#		0.509	< 0.001#

CT: choroidal thickness; IOP: intraocular pressure; CCT: central corneal thickness

* Significant difference with P < 0.05

& Significant difference with P < 0.01

# Significant difference with P < 0.001

**Table 5:** The associations of choroidal thickness with SLEDAI score and HCQ use in JSLE patients

	Average CT			Subfoveal CT			Average temporal CT			Average nasal CT	
	r	P		r	P		r	P		r	P
SLEDAI score	-0.059	0.702		-0.052	0.733		-0.051	0.739		-0.040	0.793
Cumulative HCQ dose	0.123	0.423		0.124	0.419		0.052	0.736		0.126	0.409
Duration of HCQ use	0.171	0.261		0.175	0.250		0.118	0.442		0.143	0.347

### The correlations between CT and laboratory findings

Furthermore, we also explored the potential correlations between CT parameters and various laboratory findings in JSLE patients (Table 6). The average macular CT in JSLE was negatively correlated with IL-6 and IL-10 (r=-0.337, P = 0.024, r=-0.340, P = 0.023, respectively), but showed no significant correlations with other laboratory results (all P > 0.05). Similar results were identified in the analysis of subfoveal and average temporal CT. With regard to average nasal CT, significant correlation was identified with IL-6 (r=-0.295, P = 0.049), no significant correlations were observed with any systemic laboratory findings (all P > 0.05).

**Table 6:** The associations between choroidal thickness and laboratory tests in JSLE patients

	Average CT			Subfoveal CT			Average temporal CT			Average nasal CT	
	r	P		r	P		r	P		r	P
CRP	-0.128	0.402		-0.182	0.231		-0.114	0.457		-0.105	0.492
ESR	0.074	0.631		-0.011	0.941		0.072	0.638		0.072	0.637
ALT	0.036	0.815		-0.066	0.669		-0.010	0.950		0.126	0.408
AST	0.083	0.589		0.079	0.607		0.052	0.733		0.176	0.247
Scr	0.033	0.828		-0.072	0.636		-0.010	0.946		0.068	0.655
CK	-0.128	0.402		0.167	0.273		0.113	0.460		0.123	0.422
CK-MB	0.134	0.841		0.195	0.198		0.134	0.381		0.101	0.509
ds-DNA	-0.152	0.318		-0.166	0.276		-0.133	0.385		-0.118	0.442
C3	-0.026	0.865		-0.091	0.551		-0.032	0.837		-0.036	0.815
C4	0.026	0.865		-0.031	0.840		0.050	0.746		0.019	0.903
IL-2	0.042	0.783		0.100	0.511		0.013	0.930		0.055	0.718
IL-4	-0.065	0.672		0.019	0.903		-0.068	0.657		-0.045	0.769
IL-6	-0.337	0.024*		-0.360	0.015*		-0.328	0.028*		-0.295	0.049*
IL-10	-0.340	0.023*		-0.345	0.020*		-0.365	0.014*		-0.250	0.098
TNF-α	-0.016	0.918		0.003	0.982		-0.022	0.888		0.038	0.806
INF-γ	-0.197	0.194		-0.220	0.146		-0.141	0.356		-0.181	0.233
IL-17 A	0.155	0.311		0.145	0.344		0.184	0.225		0.135	0.375

* Significant difference with P < 0.05

CT: choroidal thickness; CRP: C-reactive protein; ESR: erythrocyte sedimentation rate; ALT: alanine transferase; AST: aspartate aminotransferase; Scr: serum creatinine; CK: creatine kinase; CK-MB: creatine kinase-myocardial band; C3: complement 3; C4: complement 4

## Discussion

SLE is a chronic systemic autoimmune disease, and vascular involvement is a characteristic change in SLE pathogenesis. Histologically, circulating immune complexes are deposited in the blood vessels, especially affecting small arteries and capillaries. Dysregulation of T and B lymphocytes also contributes to vascular injury. These pathological changes of blood vessels may affect the choroid in SLE patients [20]. Recent years, OCT-derived CT has been recognized as a valuable parameter to analyze the choroidal morphological profiles [21]. Although ocular manifestations are not involved in current diagnostic criteria for SLE, they still appear to be important clues in the assessment of disease activity, since they allow direct visualization of vascular abnormalities. The incidence of SLE in China is 30 to 70 per 100,000, JSLE accounts for 10–20% of the total cases. Besides, JSLE accounts for 15–25% of children’s rheumatic disease [22, 23]. SLE is not rare in pediatric age, so accurate early screening of the ocular alterations facilitates early diagnosis and evaluation.

The macular CT values of JSLE patients without ocular involvement were compared with a healthy control group in this observational study. We found JSLE patients had decreased macular CT, suggesting a possible subclinical involvement of the choroid in JSLE. Since the vascular and connective tissue structure of the choroid grow and extend gradually, the choroid would thicken with age in childhood [24]. However, various factors could influence the physical properties of the choroidal vessels and disturb the physiologic choroidal growth. Thinner choroidal structure is assumed to be associated with hypoperfusion of the choroidal circulation. Although the onset age seemed to be very young and the duration seemed to be short, it is thought that onset age of JSLE might have been delayed and the disease duration maybe underestimated in some patients. Maybe in different stages of disease progression, the tendency of CT to change differs. Multiple lines of evidence have suggested that the choroid tends to be thicker in active phases of systemic vasculitis and autoimmune inflammatory diseases [25, 26]. However, all enrolled patients in our study were in remission phase of disease. CT may decrease with systemic inflammation, as well as systemic biological treatments. Systemic medications not only affect the microvasculature of the choroid directly, but also affect microvasculature elsewhere in the body, which result in the decreased blood supply of the choroid. Severe and repeated flares, accompanied by prolonged vasculitis, however, may lead to long-term insult and subsequent atrophy of the microvasculature, which ultimately cause the thinner choroid [25, 26]. On the contrary, Ağın et al. [27] reported the choroid was thicker in JSLE group compared to the control group at five measurement points with 750 μm intervals. Differences in patient characteristics may contribute to the discrepancy. They enrolled 21 JSLE patients and age- and sex-matched volunteer controls, however, detailed ophthalmic results were not provided. Despite age and gender, more severe myopia and longer axial length have been demonstrated to be associated with thinner CT [28]. Our study population comprised age-, sex- and axial length-matched controls to get the most consistent data and avoid bias related to these confounding factors. Besides, there are sufficient studies to confirm that there are diurnal variations in the choroid measurements [29, 30]. Therefore, we carried out OCT scans between 1 pm to 4 pm, while Ağın and colleagues didn’t emphasize the potential influence in their study design. To sum up, we would like to highlight the effect of confounding factors, further studies with more rigorous design are desiderated to explore the choroidal alterations in JSLE patients.

Previous studies also have shown evidence of subclinical alterations of the choroid in adult SLE patients. A brief literature review of OCT findings in SLE patients is shown in Table 7. Altinkaynak et al. [31] conducted a cross-sectional study involving 58 inactive adult SLE patients were and 58 healthy controls. They found CT was significantly thinner in SLE subjects as compared to the healthy controls, and the results remained the same even when the effects of age were eliminated. Dias-Santos et al. [32] also demonstrated that SLE appeared to have thinner CT, particularly in those with nephritis and receiving oral anticoagulants. This thinning choroid has been attributed to chronic inflammatory response in vascular endothelium of the choroid, along with the immune and complement deposition, as a result of decreased choroidal blood supply leading to chronic choroidal atrophy. However, Invernizzi et al. [33] revealed that SLE patients had significantly increased subfoveal CT compared with controls. Braga et al. [34] reported significantly thicker macular choroids in the eyes of SLE patients with lupus nephritis compared to eyes of SLE patients without lupus nephritis and healthy controls. Likewise, Hassan Salah et al. [35] also reported that nephritic SLE showed thickening subfoveal CT compared to non-nephritic SLE. Lee et al. [36] found SLE subjects with LN had equivalent CT measurements compared to those without LN. The discrepancy may be due to the different designs of different studies, such as differences in inclusion criteria and different cohorts at various stages of the disease. Alternatively, these different findings may also reflect the tendency of CT to change over time during different stages of JSLE. Besides, considering the inconsistency and lack of age, spherical equivalent, axial length in some of these studies (Table 7), these results should be interpreted with caution.

**Table 7:** A brief literature review of OCT findings in SLE patients

Author (year)	Study population	No. of eyes	Age (years)	Spherical equivalent (D)	Axial length (mm)	SLE duration (years)	Results
Altinkaynak et al. (2016)	58 SLE	58	44.04 ± 11.88	-0.07 ± 0.48	23.2 ± 0.57	5.43 ± 5.28	SLE had significant lower subfoveal, nasal, and temporal CT
	58 Control	58	42.33 ± 7.97	-0.24 ± 0.34	23.1 ± 0.65	NA	
Ferreira et al. (2016)	43 SLE	43	41.91 ± 12.74	-0.65 ± 1.65	NA	NA	SLE presented a thicker choroid in all measured points
	80 Control	58	39.99 ± 16.96	-0.65 ± 1.70	NA	NA	
Invernizzi et al. (2017)	60 SLE	60	42.43 ± 9.07	NA	NA	17.48 ± 9.10	SLE showed thicker subfoveal CT. No significant differences were found in CT between SLE with or without glomerulonephritis
	60 Control	60	NA	NA	NA	NA	
Ağın et al. (2019)	21 JSLE	21	16 (6–19)	NA	NA	NA	JSLE showed significant thicker macular CT
	21 Control	21	NA	NA	NA	NA	
Braga et al. (2019)	15 LN	15	45.13 ± 9.0	NA	NA	14.0 ± 6.0	SLE with LN showed significantly thicker macular CT than that of SLE without LN and healthy controls
	15 non-LN	15	45.2 ± 9.5	NA	NA	8.9 ± 6.3	
	15 Control	15	45.6 ± 9.3	NA	NA	NA	
Salah et al. (2020)	35 SLE	35	31.2 ± 9.4	NA	NA	6 (1–15)	SLE had significantly thinner macular CT. Nephritic SLE showed thickening subfoveal CT compared to non-nephritic SLE
	30 Control	30	34.3 ± 8.0	NA	NA	NA	
Lee et al. (2020)	11 non-LN	20	47 ± 12	-0.25(-2.25,-0.25)	NA	5.5(1.7,21.8)	SLE with LN had equivalent CT measurements compared to those without LN
	12 LN	23	34 ± 8.8	-1.6(-4.75,-1.6)	NA	9.0(7.3,13.0)	
Işık et al. (2021)	35 SLE	35	42.6 ± 9.2	-0.50 ± 0.76	22.9 ± 0.69	10.6 ± 4.4	SLE using HCQ and/or immunosuppressive agents had statistical thicker CT, but it reached no statistical significance
	35 Control	35	42.5 ± 7.9	-0.26 ± 0.65	22.6 ± 0.59	NA	
Forte et al. (2021)	10 SLE	20	38.87 ± 8.6	-1.54 ± 2.87	NA	10.25 ± 3.28	SLE undergoing HCQ treatment for more than 5 years showed reduced foveal CT than that of controls
	18 Control	36	41.11 ± 8.12	-0.96 ± 1.42	NA	NA	
Dias-Santos et al. (2021)	68 SLE	68	45.50 ± 12.67	-0.25(-1.0,0.25)	23.56 ± 1.00	11.0(6.25,19.00)	Macular CT showed lower values in SLE, but reached no statistical significance
	50 Control	50	52.76 ± 14.45	0.13(-0.63,1.0)	22.89 ± 0.96	NA	

Results are expressed as mean ± SD, median (interquartile range) or median (minimum-maximum), as appropriate

LN, lupus nephritis; CT, choroidal thickness; SLE, systemic lupus erythematosus; JSLE, juvenile systemic lupus erythematosus; HCQ, hydroxychloroquine; NA, not applicable.;

In our current study, CT parameters had no significant relationships with cumulative HCQ dose or duration of HCQ use. The median cumulative dose of HCQ employed for the treatment of JSLE patients was 188.5 g (range: 38.4–576). Most JSLE patients were treated for less than 5 years. The drug dose was small and the exposure time was relatively short, thus, it is not possible to clearly determine the long-time impact of HCQ on the choroid in JSLE. Ferreira et al. [37] conducted a retrospective study and reviewed the OCT profiles of adult SLE patients who were treated with HCQ screening for eye toxicity. SLE group, in the absence of HCQ-related retinopathy, presented thicker choroids in all measured points. Similarly, Işık et al. [38] found adult SLE using HCQ and/or immunosuppressive agents had statistical thicker CT than healthy controls, but it reached no statistical significance. In contrast, Ahn et al. [39] performed CT measurements in 124 HCQ-treated adult patients diagnosed with SLE or rheumatoid arthritis, which were divided according to the presence or absence of HCQ retinopathy. The results suggested significantly decreased CT in HCQ retinopathy group. Forte et al. [40] also showed reduced foveal CT in adult SLE patients undergoing HCQ treatment for more than 5 years. HCQ toxicity. The conflicting results may be related to the cumulative drug dose and duration of drug exposure, since Bulut et al. [41] indicated cumulative dose and duration of HCQ treatment had negative correlations with CT. Notably, it is hypothesized HCQ may influence CT by modulating vasoconstriction and endothelial dysfunction mediated by endothelin-1. HCQ also inhibits the production of reactive oxygen species and inflammatory cytokines, which may also contribute to increased CT in its early use. With the gradual accumulation of long-term use, HCQ probably causes retino-choroidal toxicity and leads to decreased CT [20, 42, 43]. The effect of HCQ treatment with or without retinopathy on CT needs to be further investigated. In summary, different stages of SLE may have a different repercussion on CT. There are no scientific investigations about the relationship between CT and various factors, such as medication, the presence of lupus nephritis, or other systemic comorbidities. Further studies with more rigorous selection of the study population are warranted to verify our understanding of the choroid and explore its complexity in response to systemic conditions and medications.

None of published investigations have comprehensively addressed the associations between macular CT and systemic variations in JSLE, which are critical hallmarks of disease activity and severity. We simultaneously analyzed a selection of laboratory tests in JSLE group. No significant correlations were observed between CT and hepatic, renal and cardiac biomarkers, since most JSLE patients had no systemic symptoms at the time of OCT examinations. Serum C3, C4, and anti-dsDNA antibody are important indices in SLEDAI-2 K scoring system, correlation analysis between CT and these biomarkers did not yield significant difference. Previous studies have highlighted abnormal regulation and impaired differentiation of T cells as a major characteristic in SLE pathogenesis, especially the imbalance in Th cells and Treg cells. Systemic upregulation of inflammatory cytokines in SLE patients has been postulated in previous studies [44]. In our present study, the plasma levels of Th1 (IL-2, IFN-γ, TNF-α), Th2 (IL-4), Th17 (IL-6, IL-17 A), and Treg (IL-10) cytokines were also analyzed in JSLE patients. Increased cytokines may predispose the local choroidal environment to a proinflammatory status. Furthermore, we found the average macular, temporal and subfoveal CT in JSLE patients were negatively correlated with IL-6 and IL-10 expressions, indicating that choroidal pathologies might be associated with the systemic cytokine profiles. Several researches have evaluated the role of different cytokines, such as IL-6, IL-10, IFN-γ, or TNF-α, as predictors of clinical activity in SLE. Cavalcanti et al. [45] have indicated increased IL-6 and IL-10 were significantly associated with disease activity in childhood-onset SLE. Further longitudinal studies with larger cohorts will be essential to determine whether different CT parameters can be adjunctive tools to monitor the disease activity or predict the immunological inflammatory status.

Several limitations affected this preliminary study. First of all, this was a cross-sectional observational study of the inactive phase of JSLE. Longitudinal studies exploring the choroidal alterations over time in JSLE will lead to deeper understanding of the characteristics of different periods. Second, the study participants were not naive in terms of pharmacological therapy, and they have achieved remission at the time of enrollment. Therapeutic drugs commonly used in JSLE (including corticosteroids and various immunosuppressive agents) can affect vascular integrity and cause ocular damage, but we did not consider the role of these drugs in JSLE in detail, such as the cumulative doses and duration of drug exposure. Third, OCT images only provided information of specific place and small area. CT measurements were performed manually by one experienced operator. Its reproducibility and reliability might be questioned. Nevertheless, previous studies have demonstrated high interobserver correlation, repeatability and reproducibility in CT measurements. Development of automated choroidal measurement technique across the full wide-field area is required to provide more convincing results. Moreover, JSLE participants were at different stages of the disease, subgroup analysis was not performed due to limited sample size. Our results definitely need further confirmation from larger and standardized clinical studies.

## Conclusion

JSLE patients without ocular involvement may have significant variations in choroidal thickness at the macular area. Choroidal alterations might be associated with the systemic cytokine profiles in JSLE.

## Data Availability

All data generated or analyzed during this study are included in this published article.

## List of Abbreviations

SLE: systemic lupus erythematosus
FFA: fundus fluorescein angiography
ICGA: indocyanine green angiography
OCT: optical coherence tomography
EDI: enhanced depth imaging
CT: choroidal thickness
JSLE: juvenile systemic lupus erythematosus
SLICC: Systemic Lupus International Collaborating
SLEDAI-2k: Systemic Lupus Erythematosus Disease Activity Index 2000
BCVA: best-corrected visual acuity
IOP: intraocular pressure
CCT: central corneal thickness
CRP: C-reactive protein
ESR: erythrocyte sedimentation rate
ALT: alanine transferase
AST: aspartate aminotransferase
Scr: serum creatinine
CK: creatine kinase
CK-MB: creatine kinase-myocardial band
C3: complement 3
C4: complement 4
HCQ: hydroxychloroquine
